# Identifying Challenges and Solutions to Early Childhood Education and the Perceived Importance of Outdoor Time: A Mixed Methods Approach in a Socioeconomically Diverse Population

**DOI:** 10.3390/ijerph20247166

**Published:** 2023-12-12

**Authors:** Maria B. Butcher, Magdalena K. Haakenstad, Carolyn J. Noonan, Amber L. Fyfe-Johnson

**Affiliations:** Elson S. Floyd College of Medicine, Washington State University, Spokane, WA 99202, USA; maria.butiu@wsu.edu (M.B.B.); m.haakenstad@wsu.edu (M.K.H.);

**Keywords:** outdoor preschool, early childhood education, mixed methods, preschool, nature

## Abstract

The current literature supports the positive relationship between time in nature and the improvement in children’s health and identifies early childhood education (ECE) settings as an avenue for intervention. Unfortunately, access to both outdoor time and ECE opportunities is lower in communities facing economic adversity. Efforts are needed to identify the best approaches to incorporate outdoor time in ECE settings, especially in communities facing socioeconomic adversity. The objectives of this research were to use a mixed methods approach to identify (1) barriers and solutions to the integration of outdoor time in ECE settings, (2) if outdoor time is a priority in ECE settings compared to other ECE priorities, and (3) how socioeconomic status influences ECE priorities and barriers for outdoor time, and health outcomes. Fourteen focus groups were conducted (*n* = 50) in the United States (US) with participants from three stakeholder groups: outdoor educators, parents of children attending outdoor preschool, and community members with children. Participants completed a survey (*n* = 49) to evaluate demographics, views on ECE and outdoor time, and health characteristics. Exploratory analyses of F as an effect modifier were conducted. The survey results showed that parents prioritized social and emotional learning and outdoor time when selecting an ECE setting for their child. The barriers identified include financial challenges and the limited availability of ECE programs. The solutions discussed included increased availability and financial support. Low income was correlated with higher rates of anxiety and increased outdoor time was a potential protective factor. These insights inform interventions to enhance outdoor time in ECE settings, with the goal of reducing disparities and promoting children’s overall health.

## 1. Introduction

A growing body of literature supports positive associations between outdoor time and the health of children [1]. The advantages derived from children’s exposure to nature and the outdoors are manifold, encompassing increased physical activity [2,3,4,5], improved mental health [6], stress reduction, and support for overall child development [7]. Unfortunately, many children and families lack access to outdoor spaces, particularly those facing financial adversity and those from other marginalized communities [8,9,10,11,12]. In addition, the distribution of green spaces and access to nature are inequitable, with poverty being associated with greater distances to parks and fewer green spaces in urban and suburban areas [13]. Like outdoor time, high-quality early childhood education (ECE) can provide a myriad of benefits to children by improving academic success, socioemotional skills, and health-related outcomes [14,15,16,17]. Like the inequitable distribution of outdoor spaces, high-quality ECE is scarce in low-socioeconomic status communities [18,19]. Families in these communities bear a disproportionate burden of chronic illnesses and face many health inequities including higher levels of psychological stress and diminished well-being [20,21]. The repercussions of stress on child development, as well as on physical and mental health, are both immediate and long-lasting [7,22,23,24]. In addition to the lack of access to nature and ECE, impoverished communities also grapple with inadequate resources to support their health [25,26,27,28,29] and limited educational and employment opportunities [27]. Moreover, children from marginalized families encounter obstacles to academic success and lower future earning potentials [28,29], thereby exacerbating intergenerational socioeconomic, racial, and health inequities [30]. Policy-level changes for low-income communities necessitate research efforts to (1) characterize barriers to ECE in these communities, and (2) determine whether outdoor time is a priority for those facing adversity.

Another key risk factor for child health outcomes is the health of adults in their immediate community [31,32,33,34]. Children who have parents or caregivers with lower mental health are more likely to both have poorer mental, physical, and behavioral health, and be living in a low-income family [31,32]. Children with parents in very good or excellent health are nearly four times more likely to also have very good or excellent health compared to their counterparts with parents who are not in very good or excellent health [35]. Teachers play a crucial role in children achieving academic, social, and emotional milestones [36,37]. High teacher stress and burnout are associated with lower academic performance and social adjustment in their students [38], whereas greater teacher engagement in their work predicts higher student achievement scores [34,38,39]. Given the high correlation between child health and parent, caregiver, and teacher health [34,35,40], it is important to consider educator health in ECE settings both for the health of the individual teacher and for the children in their classrooms.

Landmark studies such as the Perry Preschool Project have supported the use of ECE as an opportunity for intervention for low-income children facing adversity and as a mechanism to improve academic, economic, and health outcomes in childhood and adulthood [41]. Head Start, a large-scale federal preschool program that prioritizes low-income families, has also shown improvements in many facets in childhood and beyond, including high school completion and health outcomes [42]. Nonetheless, neither the Perry Preschool Project nor Head Start made outdoor time a priority in addressing health inequities in early childhood. The natural environment has the potential to act as both a protective factor for many health outcomes and a health-promoting space in childhood [7,20]. Similarly, high-quality ECE benefits children, especially those at high-risk of poor academic, health, and financial outcomes [43,44,45]. Thus, advocating for equitable access to nature for low-socioeconomic status children by integrating outdoor time into high-quality ECE settings holds promise to lessen the consequences of early life adversity and improve health, both in childhood and later into adulthood [46].

Although previous research has shown great benefits of outdoor time in childhood, children today are spending less time outside than they had in decades prior [47]. No studies to date have identified barriers and solutions to both outdoor time and ECE in low-income communities using a community-based participatory research framework. Interventions designed for majority groups often do not yield the same results when applied to culturally, socioeconomically, and racially diverse groups [48,49,50]. Thus, it is critical that interventions designed to address community challenges incorporate the voices and perspective of the communities they aim to serve. Accordingly, the objectives of this study were to use a mixed methods methodology to identify (1) barriers and solutions to the integration of outdoor time in ECE settings, (2) if outdoor time is a priority in ECE settings compared to other ECE priorities (e.g., social and emotional learning), and (3) how socioeconomic status influences ECE priorities and barriers for outdoor time, and mental health outcomes.

## 2. Methods

### 2.1. Theoretical Framework

This study was theoretically guided by a research framework developed by the National Institute on Minority Health and Health Disparities at the National Institutes of Health [51]. The framework includes a matrix prioritizing domains of influence and levels of influence, all of which affect populations experiencing health disparities. For this project, we prioritized the intersection of an individual-level influence and the physical/built environment domain of influence. The framework informed the process of defining both the research questions and consequently the focus group (FG) questions that aimed to identify barriers and solutions to the physical/built environment.

Additionally, this research design was guided by the principles of community-based participatory research. Incorporating voices of the community is critical to the fundamental premise of community-based participatory research [52], since nuances of cultural norms in the communities affected by inequity require the adaptation of interventions to reflect the needs and priorities of the community in question [53]. Considering existential perspectives also creates an equity-based approach to research with numerous resultant benefits: deeper understanding of the challenges faced, potential to identify root causes and structural factors contributing to inequity, and avoidance of harmful unintended consequences [52,53].

### 2.2. Setting and Participants

In 2010, there were fewer than 100 outdoor preschools across the country. The number of programs has increased rapidly, with an estimated 800 programs now available [54]. Outdoor preschools were first licensed in Washington state in 2019, following a 4-year pilot program. They have increased in popularity and accessibility since then, and there are currently over 50 licensed outdoor preschools state-wide [54]. Outdoor preschools, or nature-based ECE programs, have nature as both a setting and object of study. Children enrolled in these programs spend an average of 80% of the school day outside [54].

This study was conducted in partnership with an exclusively outdoor preschool in the Seattle, Washington (US), metropolitan area. Several unique features of this preschool made it an ideal partnership. First, it is one of the largest outdoor preschools in the Seattle area and enrolls ~300 families annually. Second, their social equity model reserves half of the enrollment spots for children from families qualifying for free or reduced tuition, thus facilitating the enrollment of a diverse socioeconomic population. All families interested in enrolling submit applications and are selected on a first-come, first-served basis.

The Washington State University Institutional Review Board approved this study, and all participants provided informed consent. All participants were required to be a minimum of 18 years old and able to provide informed consent in English. Three key stakeholder groups were recruited, with broad inclusion and exclusion criteria. The first stakeholder group was parents with children attending outdoor preschool. Parents were selected if they had a preschooler between the ages of 3 and 5 enrolled at the preschool with which the study team was partnered. Families with foster children were excluded due to legal considerations of foster children. The second group was community members with children. This group was selected from parents who made use of a local community center. The third stakeholder group comprised outdoor educators, including staff, administrators, or leaders at the partnered outdoor preschool or similar programs.

Participants fitting one of the following criteria were recruited for enrollment in this convergent parallel mixed methods study: parents of a child enrolled in outdoor preschool, outdoor preschool educators, and parents in the target community of interest. Following study flyer distribution through email and direct communication with the ECE community network in King County, participants (*n* = 50) were enrolled in the study between January and April 2022. The number of participants enrolled was informed by the expertise of study team members with extensive experience in qualitative research methods and was targeted to be sufficient for thematic saturation. All enrolled participants participated in a single FG; 49 participants completed the health needs assessment survey. All participants received a USD 50 gift card for their participation.

### 2.3. Procedures

#### 2.3.1. Focus Groups (Qualitative)

From April to June 2022, the research team conducted 14 FGs. Three FGs were held in person and 11 virtually over Zoom based on the availability of participants. The FG moderators (the second and last authors) used a systematic and comprehensive protocol with 10 open-ended questions and follow-up probes to examine challenges and facilitators to ECE. Three of the FG questions were specific to ECE and are the focus of this manuscript. Participants were also asked to reflect and share their perspectives on the importance of outdoor time for children. All FGs were audio-recorded, and participants remained anonymous during their study participation. The size of the FGs ranged from 3-10, consistent with guidance for thematic saturation [55], with one exception due to last-minute scheduling conflicts that resulted in a single participant being present for an FG. FGs lasted approximately 1 h. Quantitative data were collected using a health needs assessment survey developed by the study team. Study participants completed the survey using REDCap following the FG, and the surveys took approximately 20 min to complete.

#### 2.3.2. Measures (Quantitative)

##### Demographics

A health needs assessment survey designed by the study team was used to collect demographic information that included age, race, ethnicity, sex assigned at birth, gender identity, current relationship status, employment status, and highest terminal degree. Study participants were also asked to report the range of their annual household income.

##### Physical and Mental Health

General self-reported health was assessed using a single question from the SF-12 survey [56], and participants rated their health as poor, fair, good, very good, or excellent. Participants also self-reported height and weight and their body mass index (BMI) was calculated using the following formula [57]:

$$BMI=\frac{weight\left(kilograms\right)}{{height\;(meters)}^{2}}$$

Categorization of overweight (BMI 25–29.9) and obesity (BMI > 30) was performed according to the Centers for Disease Control and Prevention guidelines [57]. Anxiety and depression were assessed using the Generalized Anxiety Disorder 7-item (GAD-7) scale [58] and a modified version of the Patient Health Questionnaire-9 (PHQ-9) [59], respectively. The GAD-7 is a reliable and valid tool for screening for generalized anxiety disorder [58]. The PHQ-9 is a reliable and valid screening measure to assess the risk of depression [59]. The PHQ-9 was modified by omitting a single question regarding suicidality due to limited study resources available to provide appropriate support if suicidal ideation was identified. Participants reported actual time spent outdoors in minutes on weekdays and weekends, and weekly outdoor time was calculated as a sum of weekday and weekend time. Using questions generated by the study team, participants were asked to identify (1) how important 10 common preschool activities were to prepare their children for kindergarten (learning ABCs and numbers, outside playtime, etc.), and (2) barriers to accessing preschool education in their communities from a 10-item list (limited hours, too expensive, etc.).

### 2.4. Data Analysis

#### 2.4.1. Quantitative

Means and standard deviations were calculated as descriptive statistics for continuous variables, and categorical variables are described using counts and frequencies. Most survey results were stratified by high- (greater than or equal to USD 90,000) and low- (below USD 90,000) income. The categories were informed by the median income in the county of interest (USD 102,620 per year) compared to the median income of Washington state (USD 80,319 per year) [60]. Of note, these values are also utilized by our community partner in their determination of eligibility for free or reduced tuition. Descriptive statistics were categorized based on educator status (educator vs. non-educator) and three income categories (<USD 50,000, USD 50,000–90,000, and <USD 90,000) to assess a more granular categorization of income. These thresholds were chosen due to (1) small sample sizes preventing further breakdown by income, and (2) historical cutoffs used by ECE settings in the Seattle metropolitan area to determine free or reduced tuition eligibility. All analyses were conducted using Stata 17 [61].

#### 2.4.2. Qualitative

Audio recordings of the FGs were transcribed using rev.com (accessed on 15 May 2023) [62] and uploaded to Dedoose (version 9) [63], a software used for mixed methods data analysis. The codebook was developed by the first and second authors using a systematic and iterative approach. First, coders independently reviewed transcripts and recorded themes and subthemes. Then, findings were examined during bi-weekly meetings to discuss and clarify findings and ensure alignment with the scope of the project. Finally, the coders worked together to refine the codebook and consolidate and define the major themes and subthemes. The themes identified emerged from thematic analysis of the FG transcripts and are analyzed in this manuscript. Thematic saturation was reached when no new codes emerged and all transcripts had been coded [64,65]. A Dedoose inter-rater reliability test was conducted, which revealed an 84% Cohen’s kappa value score, suggesting excellent agreement between the two coders [66].

## 3. Results

### 3.1. Demographic Characteristics (Table 1)

Study participants predominantly identified as Caucasian (73% of low-income and 79% of high-income) and female (89% of low-income and 100% of high-income). All high-income participants reported being married or partnered compared to only 44% of those in the low-income category, and 68% completed a post-graduate or professional degree compared to 19% of those in the low-income category.

**Table 1:** Demographic characteristics (*n* = 46).

	Annual Household Income ^1^			
	<USD 90,000 (*n* = 27)		USD 90,000+ (*n* = 19)	
**Age, mean years (SD)**	31	(6)	37	(4)
**Race, *n* (%)**				
Paricipants of color	7	(27%)	4	(21%)
Caucasian	19	(73%)	15	(79%)
**Sex assigned at birth, *n* (%)**				
Male	3	(11%)	0	(0%)
Female	24	(89%)	19	(100%)
**Current married or partnered, *n* (%)**	12	(44%)	19	(100%)
**Currently employed, *n* (%)**	21	(78%)	15	(79%)
**Completed education, *n* (%)**				
High school/GED	6	(22%)	1	(5%)
Technical/vocational, associate, or bachelor’s degree	16	(59%)	5	(26%)
Post-graduate or professional degree	5	(19%)	13	(68%)

^1^ *n* = 3 participants missing income.

### 3.2. Qualitative and Mixed Methods (Table 2)

Table 2 summarizes the FG questions, FG themes identified from the analysis of FG transcripts (qualitative), survey results (quantitative), and an overall mixed methods summary.

**Table 2:** Focus group questions and identified themes, quantitative results, and mixed methods summary.

Focus Group Questions	Focus Group Themes (Qualitative)	Survey Results (Quantitative)	Mixed Methods Summary
**Perceived Importance of Outdoor Time**	Outdoor time improves childhood physical health. Outdoor time improves childhood mental health. Children do not spend enough time outdoors.	100% of participants in both income categories indicated that outdoor time in ECE settings was either “very” or “somewhat important”. ^1^ No differences in importance of outdoor time between educators and non-educators. ^1^	**General:** alignment between qualitative and quantitative results. Both qualitative and quantitative results revealed that participants find great value in outdoor time for children, and it is one of the top priorities in ECE settings.
**Challenges to ECE**	Financial challenges. Availability (lack of transparency of childcare system, limited spots and hours, lack of quality childcare, etc.). Time constraints (work schedules). Discomfort and safety. Staff shortages.	ECE too expensive (low-income: 85%, high-income: 74%). ^2^ Limited ECE availability (low-income 78%, high-income: 84%). ^2^ Limited ECE hours (low-income: 70%, high-income 63%). ^2^	**General:** alignment between qualitative and quantitative results. Both qualitative and quantitative results revealed alignment in challenges to ECE: financial challenges and availability (general availability and hours).
**Solutions to ECE**	Financial support. Educator compensation. Transparency of the childcare system.	N/A	Solutions to ECE were only assessed qualitatively.

^1^ Table 3. ^2^ Table 4.

#### 3.2.1. Focus Group Question 1: Perceived Importance of Outdoor Time (Table 2)

When asked how much time children should spend outdoors, the answers were overwhelmingly positive and varied from 2 h to “as much as possible”. Some participants expressed that there is no amount of outdoor time that is best for all children, but rather it varies based on individual needs. Participants consistently articulated concerns that children do not spend enough time outdoors and that it would be helpful to promote outdoor play in ECE settings. The positive outcomes of outdoor time were discussed and fell into three categories. First, there was consistent agreement that outdoor time improves childhood physical health given that when kids are outside, they are physically active, become stronger, burn off energy, explore their physical limits (e.g., how high they can climb on a tree), have fewer illnesses (e.g., colds), and may have improved tolerance to allergens. Second, participants reported that outdoor time improves childhood mental health. Common themes included time outdoors to promote learning, improve resilience, encourage independence, gain emotional intelligence, overcome obstacles, deal with discomfort, and bring joy. Despite consistent agreement among participants that outdoor time is beneficial for children, there was consistent concern that children do not spend enough time outdoors. Participants reported that the value system of the outdoors has changed across generations, and that when they were children, being outside without consistent adult supervision was nearly universal. Children would play outside in neighborhoods with other children in the community for extended periods of time. In contrast, current societal norms leave parents feeling worried about leaving their children alone in parks or in their neighborhoods. The primary driver of this fear is pervasive societal expectations and norms that suggest that it is not acceptable or appropriate to leave children alone without parental supervision.

#### 3.2.2. Focus Group Question 2: Challenges to ECE (Table 2)

Focus group participants were asked to reflect on challenges to ECE. The most common answer was financial challenges in paying for tuition. Multiple people shared that in a dual-income household, one parent’s salary may be almost entirely devoted to covering childcare/preschool costs. Some expressed that without financial support in the form of free/reduced tuition at certain programs, it would be impossible for families to access their preferred childcare/preschool resource. Challenges regarding availability of ECE was the next most consistently discussed barrier. Participants reported that it is difficult to find information about preschool programs available in their communities. Some participants expressed relying on word-of-mouth to gather information about childcare/preschool, while others described using the internet or reaching out to many programs directly, with variable success in gathering the information they wanted. This lack of transparency in the childcare system further challenged parents attempting to make an informed choice for their children. Similarly, some parents expressed frustration with the limited spots available for certain programs. Long waiting lists were discussed as another barrier to accessing childcare/preschool. Some participants said they would prefer for their children to attend outdoor preschool but the limited hours they provide made them choose a standard daycare setting, although they find outdoor time there insufficient. Another related topic was the lack of quality childcare. This varied among participants, as some discussed quality in terms of outdoor time offered to children, while others valued the racial and ethnic diversity of childcare/preschool members. In addition to the challenges of covering the cost of attendance and availability, participants often talked about time constraints for parents when preschool programming was not full day coverage.. They expressed how variable pick-up and drop-off times for partial day coverage were often prohibitive.

Discomfort evolved into a theme that incorporated ideas beyond those related solely to outdoor schools to encompass any discomfort of being out in nature. Participants discussed weather (both wet and cold), getting dirty, insects, and other elements of nature that created a feeling of discomfort, both for children and adults. Interestingly, the discomfort discussed was almost universally seen in a positive light: children learned more about themselves and their environment when they were faced with and overcame uncomfortable elements. Participants discussed safety and emergency plans both in terms of injury concerns and the inconvenience of outdoor school cancelations due to inclement and unsafe (e.g., smoke during summer months) weather being unpredictable.

During FGs with outdoor educators, staff shortages emerged as a recurring concern. Educators cited discomfort with extended outdoor hours as a deterrent for seeking employment in outdoor preschools. Additionally, the absence of enclosed spaces necessitates higher educator-to-child ratios compared to indoor preschools. The COVID-19 pandemic disrupted the job market, prompting educators to explore more stable career options. Consequently, the demand for outdoor educators has surged, posing challenges in recruiting and retaining a qualified workforce.

#### 3.2.3. Focus Group Question 3: Solutions to ECE (Table 2)

Mirroring the responses to “Challenges to ECE”, the most discussed solution to ECE was financial support for families to cover the cost of attendance. Ideas included government funding of preschool/childcare programs, as well as free/reduced tuition based on household income. Another theme that emerged was improving educator compensation, which would also help overcome the staffing shortages. Participants suggested several ways to improve the transparency of the childcare system, either through the compilations of available childcare resources or the introduction of a rating system so families could share their experiences with different programs.

### 3.3. Quantitative Results

#### 3.3.1. Importance of Activities to Prepare Children for Kindergarten (Table 3)

Table 3 summarizes results for the importance of ECE activities for getting children ready for kindergarten. The highest priorities for all respondents in both income categories were social and emotional learning and outside playtime. Social and emotional learning was indicated to be “very important” for 90–94% of low-income participants (*n* = 25) as an activity that would prepare children for kindergarten, and the remaining 6–10% (*n* = 2) indicated it was “somewhat important”. Results were similar for high-income participants. Outdoor time was ranked “very important” by 88–90% of low-income participants (*n* = 24) as an activity that would prepare children for kindergarten. The remaining 10–12% (*n* = 3) indicated that outdoor time was “somewhat important”. Results were similar for high-income participants. For both social and emotional learning and outdoor time, 100% of participants in both income categories indicated that these activities were either “very” or “somewhat important”.

**Table 3:** Importance of ECE activities for getting children ready for kindergarten (*n* = 46).

	Annual Household Income ^1^							
	<USD 90,000				USD 90,000+			
Activity ^2^	Non-Educator (*n* = 10)		Educator (*n* = 17)		Non-Educator (*n* = 17)		Educator (*n* = 2) ^3^	
**Social and emotional learning, *n* (%)**								
Somewhat important	1	(10%)	1	(6%)	2	(12%)	0	(0%)
Very important	9	(90%)	16	(94%)	14	(88%)	2	(100%)
**Outside playtime, *n* (%)**								
Somewhat important	1	(10%)	2	(12%)	2	(12%)	0	(0%)
Very important	9	(90%)	15	(88%)	15	(88%)	2	(100%)
**Make believe play, *n* (%)**								
Somewhat important	2	(20%)	3	(18%)	3	(18%)	0	(0%)
Very important	8	(80%)	14	(82%)	14	(82%)	2	(100%)
**Story time, *n* (%)**								
Somewhat important	2	(20%)	4	(24%)	4	(24%)	2	(100%)
Very important	8	(80%)	13	(76%)	13	(76%)	0	(0%)
**Music, *n* (%)**								
Not very important	0	(0%)	0	(0%)	2	(12%)	0	(0%)
Somewhat important	2	(20%)	8	(47%)	7	(41%)	1	(50%)
Very important	8	(80%)	9	(53%)	8	(47%)	1	(50%)
**Arts and crafts, *n* (%)**								
Not very important	0	(0%)	1	(6%)	1	(6%)	0	(0%)
Somewhat important	3	(33%)	8	(47%)	5	(29%)	2	(100%)
Very important	6	(67%)	8	(47%)	11	(65%)	0	(0%)
**Science activities, *n* (%)**								
Not very important	0	(0%)	1	(6%)	2	(12%)	0	(0%)
Somewhat important	6	(60%)	7	(41%)	10	(59%)	1	(100%)
Very important	4	(40%)	9	(53%)	5	(29%)	0	(0%)
**Learning ABCs and numbers, *n* (%)**								
Not very important	2	(20%)	2	(12%)	3	(18%)	2	(100%)
Somewhat important	5	(50%)	12	(71%)	7	(41%)	0	(0%)
Very important	3	(30%)	3	(18%)	7	(41%)	0	(0%)
**Inside playtime, *n* (%)**								
Not important	0	(0%)	2	(12%)	0	(0%)	1	(50%)
Not very important	3	(30%)	6	(35%)	2	(12%)	0	(0%)
Somewhat important	4	(40%)	7	(41%)	9	(53%)	1	(50%)
Very important	3	(30%)	2	(12%)	6	(35%)	0	(0%)
**Nap time, *n* (%)**								
Not important	1	(10%)	0	(0%)	1	(6%)	0	(0%)
Not very important	3	(30%)	3	(18%)	5	(29%)	1	(50%)
Somewhat important	2	(20%)	9	(53%)	9	(53%)	1	(50%)
Very important	4	(40%)	5	(29%)	2	(12%)	0	(0%)

^1^ *n* = 3 participants missing income; ^2^ not important and not very important were also possible response options but not selected; ^3^ limited interpretability given small sample size.

#### 3.3.2. Challenges and Solutions to ECE (Table 4)

The financial challenges discussed in FGs were mirrored by the results of the survey, with 85% of participants in the low-income category and 74% of participants in the high-income category ranking the cost of preschool as a barrier in their community (Table 4). Limited hours provided by ECE settings was also noted in quantitative results, with 70% of low-income and 63% of high-income participants reporting limited hours as a barrier to preschool in their community (Table 4). When limited availability was assessed, 84% of high-income participants reported this as a significant barrier, as well as 78% of low-income participants (Table 4).

Several additional barriers to obtaining ECE were identified in the surveys that were not discussed in FGs. These included transportation as a barrier for pick up and drop off (63% for low-income and 47% for high-income), inconvenient locations (48% for low-income and 32% for high-income), language or other cultural barriers (30% for low-income and 16% for high-income), and difficulty finding a good fit for their child (26% for low-income and 26% for high-income) (Table 3). “Too much paperwork” (7% for low-income and 11% for high-income) and “too impersonal” (7% for low-income and 5% for high-income) were reported as barriers for less than 12% of participants (Table 4).

Although this was commonly discussed in FGs, quantitative data revealed that only 67% of low-income and 58% of high-income participants believed there is not enough tuition assistance available, despite 85% of low-income and 74% of high-income participants identifying cost as a barrier (Table 4).

**Table 4:** Barriers to receiving ECE for children in participants’ community (*n* = 46).

	Annual Household Income ^1^			
Barrier	<USD 90,000 (*n* = 27)		USD 90,000+ (*n* = 19)	
Limited hours, *n* (%)	19	(70%)	12	(63%)
Limited availability, *n* (%)	21	(78%)	16	(84%)
Not enough tuition assistance, *n* (%)	18	(67%)	11	(58%)
Too expensive with or without tuition assistance, *n* (%)	23	(85%)	14	(74%)
Too hard to find one that is a good fit for my child, *n* (%)	7	(26%)	5	(26%)
Too much paperwork, *n* (%)	2	(7%)	2	(11%)
Too impersonal, *n* (%)	2	(7%)	1	(5%)
Language or other cultural barriers, *n* (%)	8	(30%)	3	(16%)
Inconvenient locations, *n* (%)	13	(48%)	6	(32%)
Transportation is a barrier for pick-up and drop-off, *n* (%)	17	(63%)	9	(47%)

^1^ *n* = 3 participants missing income.

#### 3.3.3. Physical and Mental Health Outcomes (Table 5)

Outdoor educators spent more time outside per week (mean (SD) = 1907 (544) min) than other study participants (mean (SD) = 543 (341) min). Given the great differences in weekly outdoor time between parents and outdoor educators, physical and mental health outcomes were analyzed separately for non-educators and outdoor educators (Table 5). This was performed to examine whether outdoor time acts as a potential protective factor, modifying negative health effects correlated with low-income status. For non-educators, an income below USD 50,000 was correlated with worse self-reported health, higher BMI, and greater anxiety and depression scores. Low-income non-educators also reported less outdoor time per week than higher-earning categories, with an average of 371 (SD 344) min compared to 527 (SD 329) min in middle-income earners and 589 (SD 351) min in high-income earners. As expected, outdoor educators in all income categories reported spending substantially more time outdoors than non-educators. On average, outdoor educators with an annual household income below USD 50,000 reported similar self-reported health, BMI, and anxiety and depression scores than their higher-earning counterparts. Approximately 55% of participants with an income <USD 50,000 reported having overweight or obesity, compared to only 17% of those earning USD 50,000–90,000 and 0% of those earning >USD 90,000. Anxiety scores nearly doubled in high-income (>USD 90,000) outdoor educators compared to those earning <USD 50,000 or USD 50,000–90,000, but the sample size in the high-income group was small.

In summary, higher income is generally correlated with better health outcomes and outdoor time may be a protective factor given most educators were in the lowest income category. Individuals with higher incomes had better self-reported health, lower BMI, and a lower prevalence of overweight or obesity. Additionally, anxiety and depression scores tended to be lower among individuals with higher incomes. However, these trends were not consistent for educators, who had a vastly greater weekly outdoor time compared to non-educators, indicating that the relationship between income and health outcomes may vary depending on the amount of outdoor time.

**Table 5:** Physical and mental health outcomes stratified by annual household income among non-educators (*n* = 29) and educators (*n* = 20).

	Income < USD 50,000 (*n* = 4) ^6^	Income USD 50,000–90,000 (*n* = 6)	Income USD 90,000+ (*n* = 17)
**Non-educators**	**(*n* = 4)**	**(*n* = 6)**	**(*n* = 17)**
Age, years (SD)	36 (5)	34 (4)	38 (3)
Weekly outdoor time, mean mins (SD) ^1^	371 (344)	527 (329)	589 (351)
Very good or excellent health, *n* (%) ^2^	1 (25%)	5 (83%)	14 (82%)
Body mass index, mean kg/m^2^ (SD)	45 (12)	25 (5)	26 (6)
Overweight or obesity, *n* (%) ^3^	4 (100%)	1 (17%)	6 (35%)
GAD-7 (anxiety), mean score (SD) ^4^	11.8 (8.1)	3.2 (1.5)	5.5 (4.1)
PHQ-9 (depression), mean score (SD) ^5^	11.8 (7.3)	2.3 (2.7)	3.7 (4.5)
**Educators**	**(*n* = 11)**	**(*n* = 6)**	**(*n* = 2) ^6^**
Age, years (SD)	30 (7)	27 (6)	32 (11)
Weekly outdoor time, mean mins (SD)	2021 (441)	1745 (340)	1770 (1570)
Very good or excellent health, *n* (%)	5 (45%)	2 (33%)	2 (100%)
Body mass index, mean kg/m^2^ (SD)	25 (5)	23 (6)	20 (1)
Overweight or obesity, *n* (%) ^3^	6 (55%)	1 (17%)	0 (0%)
GAD-7 (anxiety), mean score (SD) ^4^	6.3 (3.6)	6.5 (3.5)	12.5 (6.4)
PHQ-9 (depression), mean score (SD) ^5^	4.1 (3.2)	6.2 (3.5)	6.0 (1.4)

**Abbreviations:** SD = standard deviation, kg = kilogram, m = meter, Generalized Anxiety Disorder-7 = GAD-7, Patient Health Questionnaire-9 = PHQ-9. ^1^ Sum of self-reported weekend plus weekday outdoor time. ^2^ Self-reported from single question on Short Form (SF-16) survey. ^3^ Overweight = BMI ≥ 25.0 to <30.0; obesity = BMI ≥ 30.0. ^4^ GAD-7 scores range from 0 to 21 and higher scores indicate more anxiety: 0–4 = minimal anxiety, 5–9 = mild anxiety. 10–14 = moderate anxiety, ≥15 = severe anxiety. ^5^ PHQ-9 (suicidal ideation question removed, see Methods) scores range from 0 to 24 and higher scores indicate more depression. ^6^ Limited interpretability given small sample size.

## 4. Discussion

The primary aim of this study was to, in a socioeconomically diverse population, better understand the barriers and solutions families face in accessing ECE in their communities. This study also sought to describe the importance of outdoor time in ECE settings compared to traditional ECE priorities, and how socioeconomic status shapes ECE priorities and health outcomes. Collectively, these objectives provide an essential next step to identifying how interventions and policies can be designed to optimize access to both ECE and outdoor time for families and children facing financial adversity. The most common challenges to accessing ECE settings were financial challenges and limited availability. Intuitively, the most common solution to accessing ECE settings was financial support. Outdoor time was identified as a top priority for all respondents, in addition to social and emotional learning. Lastly, increased outdoor time was correlated with improved health, irrespective of income. Given that health outcomes in childhood are strongly correlated with the health status of adults in their family and local community [31,32], this result informs the potential ripple effect that healthier educators and family members can have on children.

The greatest barriers identified in this study were the high costs and limited availability of ECE programs. In addition, when outdoor educators were asked in FGs about challenges to ECE, they discussed staffing shortages due to poor compensation and the lack of stability in their profession, which was exacerbated by the COVID-19 pandemic. When stratified by income, outdoor educators predominantly fell into the low-income (<USD 50,000 annually) category. These discrepancies between the financial burden on families and the limited availability of ECE programs contrast with the lack of adequate compensation for educators and poor job stability. Together, this highlights the need for change to better meet the needs of educators, parents, and the children who benefit greatly from these programs. Given that approximately 63% of children under the age of 5 spend time in ECE settings [67] and a vast body of literature supports the benefits of high-quality ECE, it is vital that barriers to the accessibility of these programs are identified at a community level and addressed by systemic changes. Thus, state- and locally-funded ECE programs have the potential to reduce disparities in early education opportunities [68]. Universal preschool has emerged as a prominent ECE initiative aimed at providing equitable access to high-quality preschool education for all children [43]. The concept has gained momentum globally, with notable implementations in European countries [69]. Extending the public school system in the US to preschool-aged programming has been discussed in the US for decades, and efforts to establish universal preschool have been ongoing, with various states and cities implementing their own initiatives [70]. Universal preschool has been associated with numerous benefits for children, families, and society as a whole. Children enrolled in high-quality preschool programs exhibit enhanced foundational skills in areas such as literacy and numeracy, leading to improved academic readiness [71,72,73]. Additionally, preschool education fosters social and emotional development, cultivating vital skills like cooperation, empathy, and self-regulation [73]. Integrating outdoor time in a universal preschool program has the potential to address barriers such as high costs and limited availability, aligning with parents’ priorities for social and emotional learning and outdoor play. Universal preschool that incorporates outdoor time holds promise in addressing achievement gaps and promoting health, particularly for children from marginalized backgrounds, by prioritizing equal opportunities for early learning and outdoor time and the health benefits thereof.

In addition to navigating the high costs and limited availability of ECE programs, study participants with children also reported seeking out programs that prioritize offering children opportunities for growth in areas they deem important. Unexpectedly, few parents identified “learning ABCs and numbers” as a top priority for their preschool-aged children. Rather, “social and emotional learning” and “outside playtime” were ranked highest among both high- and low-income categories. It is critical when conducting community-based participatory research to understand the perspectives of individuals who have the lived experience of financial adversity [51]. Our results indicate that outdoor time is considered important by key stakeholders, regardless of socioeconomic status. Despite the recommendation in Washington state that children spend 90 min/day of outdoor time at preschool, preschool-aged children in Washington spend 30 min of outdoor time per day [74]. Thus, promoting increased outdoor time in ECE settings presents a mechanism to improve health for all children and promote health equity for marginalized pediatric communities. To achieve this, it is essential to prioritize equitable access to nature-based environments and resources for all children, regardless of their socioeconomic background. Incorporating culturally diverse and inclusive nature-based activities in the curriculum can ensure that children from different backgrounds benefit equally from outdoor learning experiences. Research has identified the need for greater inclusion and diversity in outdoor experiences; yet, there has been a lack of systematic efforts to transform the mainstream outdoor sector and foster genuine acknowledgment of human cultural diversity [75]. Addressing this lack of diversity in outdoor spaces has the potential to increase the use of and accessibility to nature for families with diverse backgrounds. In addition, providing training and support to educators, particularly those serving marginalized communities, can help address disparities in outdoor learning opportunities. Although this has been attempted through local pilot programming initiatives [76], more research and efforts are needed to evaluate the real-world impacts of these initiatives and to investigate them on a larger scale. Collaborative partnerships with community organizations and stakeholders can further enhance access to outdoor spaces and nature-based programming, contributing to health equity in ECE settings. By applying a nature-based health equity lens, ECE settings can foster equal opportunities for all children to thrive through outdoor engagement. This approach not only supports child development but also cultivates a lifelong connection with the natural world, fostering children’s well-being and environmental stewardship.

Participant health outcomes were analyzed with respect to income and educator status (Table 5) given both are known to be associated with positive health outcomes in early childhood [35,40]. Health outcomes data collected by surveying study participants revealed that lower income status was correlated with poorer health outcomes. However, this relationship was observed to be weaker among educators who spent more time outdoors compared to non-educators who spent less time outdoors. Additionally, participants in the higher-income category exhibited better health outcomes when compared to their lower-income counterparts. Thus, outdoor time has the potential to have a protective “equigenic” effect and could counteract the association between financial adversity and poor health outcomes [20,77]. Nature-based learning through direct contact with nature has been shown to improve health-related quality of life and academic outcomes in low-income and marginalized middle school-aged children [78]. This supports efforts to address barriers to outdoor time and ECE for at-risk families, as it has the potential to improve health outcomes in childhood and address systemic pediatric health inequities.

Several limitations of this study impact the generalizability of these results. First, this study was conducted in the Pacific Northwest where the relatively mild climate impacts values and views on outdoor time. Geographical location and associated weather contribute to shaping the unique perspectives of the study participants, which may be different from parents living in very hot or very cold climates, where outdoor schools are not as common or practical. Second, selection bias may also have been introduced due to recruitment in the ECE and outdoor preschool networks. Third, despite goals to recruit a diverse group of study participants, the majority of those enrolled in this study identified as White, and 93% identified as female. This gender distribution was expected as childcare and ECE continue to be predominantly female professions and societal norms ascribe child rearing to mothers. This trend is consistent when looking at a larger scale as well. According to the US Bureau of Labor Statistics, 97.4% of kindergarten and preschool educators are women [79]. Fourth, health outcomes were examined with respect to income and educator status, not outdoor time. Despite all educators reporting more outdoor time than non-educators, differences in health outcomes may be impacted by other factors. Fifth, due to the small sample size, all results are exploratory and hypothesis-generating in nature. This study was conducted on a smaller scale with the specific intention of determining (1) the feasibility for a larger-scale project, and (2) whether there was any indication that communities facing financial adversity were interested in prioritizing outdoor time in ECE settings. Based on these results, we plan to pursue a larger-scale project that will have a robust sample size with sufficient statistical power to answer the research questions of interest. This study also has a number of strengths. First, the rigorous mixed methods approach allowed for a more comprehensive and nuanced understanding of the topic studied. The qualitative results expanded upon and provided valuable insight into the trends that were observed from the analysis of quantitative data, thus enhancing the validity and depth of these findings. Second, this research incorporates community-based participatory research to include the perspectives of community members on outdoor time and ECE. The community-based participatory research philosophy centers community perspectives as the guiding force of future research objectives with the goal of designing and implementing an intervention to address identified barriers. Third, this work encompasses views from a diverse group of community stakeholders: outdoor educators, parents with children attending an outdoor preschool, and parents with children in the community.

## 5. Conclusions

This study highlights known barriers to ECE, the principal of which is cost and availability, and provides unique insight into the value parents place on outdoor time for their children in ECE settings. These results support the need for more accessible ECE opportunities with increased outdoor time. Outdoor time was identified as being beneficial, with improved health outcomes for participants with increased outdoor time, irrespective of income. Increasing outdoor time in an ECE setting has the potential to disrupt the relationship between financial adversity and poor health outcomes.

## Data Availability

The data presented in this study cannot be shared through commonly used data sharing repositories. The consent did not address broad sharing of participants data, nor the potential risks associated with broad data sharing of these types of data.
